# Microenvironmental Rigidity of 3D Scaffolds and Influence on Glioblastoma Cells: A Biomaterial Design Perspective

**DOI:** 10.3389/fbioe.2018.00131

**Published:** 2018-09-24

**Authors:** Ilaria Elena Palamà, Stefania D'Amone, Barbara Cortese

**Affiliations:** ^1^Nanotechnology Institute, CNR-Nanotechnology Institute, Lecce, Italy; ^2^Nanotechnology Institute, CNR-Nanotechnology Institute, University La Sapienza, Rome, Italy

**Keywords:** 3D scaffolds, glioblastoma, stiffness, neurospheres, microenvironment

## Introduction

Glioblastoma (GBM), or grade IV glioma, is an extremely aggressive tumor that infiltrates through the brain leaving the patient with a median survival time from 12 to 15 months (Ostrom et al., 2013). Individual aspects of the microenvironment features play a critical role on GBM cell dynamics and treatment resistance (Bellail et al., 2004; Zamecnik, 2005; Calabrese et al., 2007). Because of GBM's aggressive and invasive behavior, inhibition of GBM migration is envisaged as an important therapeutic objective (Bravo-Cordero et al., 2012; Wells et al., 2013). However, current models fail to account for the complex brain microenvironment. The demand of preclinical models that can faithfully mimic the clinical scenario may bridge the discrepancy between preclinical and clinical data and aid to develop treatments that are more effective.

The extracellular matrix (ECM) of the GBM microenvironment is constitutively composed of the polysaccharide hyaluronic acid (HA), and in a distinctive minor degree of tenascin-C, collagen IV and V, fibronectin, and laminin (Giese and Westphal, 1996; Rape et al., 2014). Also, typically with high glioma grade, the HA's cellular receptor CD44 is overexpressed, suggesting that CD44-enriched cells invade more efficiently the brain parenchyma (Bellail et al., 2004). GBM malignancy is furthermore promoted through interactions with the other aforementioned ECM components through different biochemical pathways (Sarkar et al., 2006; Lathia et al., 2012), which trigger an increase of the concentration of the non-cellular components (Bellail et al., 2004; Lathia et al., 2012). This increased density of the tumor ECM consequently increases the mechanical stiffness of the microenvironment (Ananthanarayanan et al., 2011; Wiranowska and Rojiani, 2011; Pathak and Kumar, 2012; Pedron and Harley, 2013; Kim and Kumar, 2014; Umesh et al., 2014; Heffernan et al., 2015).

In our opinion, to gain further insights into tumor invasiveness, heterogeneity and treatment resistance, we have to look more deeply at how cell behavior is influenced by matrix stiffness (a process known as mechanotaxis or durotaxis) (Lo et al., 2000; Cortese et al., 2009; Palamà et al., 2012, 2016).

## What is the influence of the 3D scaffold mechanical properties?

Notably, 2D platforms do not adequately mimic the *in vivo* tumor environment. Recent work has focused on 3D scaffolds and matrix influence on cells with different materials and cells, as reported in Table 1. However, inconsistencies on how 3D scaffold stiffness affect cell proliferation and influence drug delivery and treatment resistance have been reported in literature (Wang et al., 2014, 2016; Heffernan et al., 2015; Pedron et al., 2015; Lv et al., 2016; Palamà et al., 2017). In order to tune the mechanical properties of different materials, the most common methods are (1) altering the crosslink density and (2) changing the base polymer concentration which both influence different parameters, such as the ECM architecture, stiffness, pore size, diffusion of soluble factors of the scaffolds, and ligand density. A 3D culture platform that aims to mimic the native GBM microenvironment has also the additional requirement of containing HA. Pure HA lacks mechanical strength and the ability to promote cell adhesion due to its anionic properties (Wang et al., 2012). Moreover, it does not allow control over mechanical stiffness. These downsides have been partly overcome by using synthetic ECM polymers (Lutolf and Hubbell, 2005; Seliktar, 2012). One semi-synthetic material, predominantly used to independently tune the stiffness of the scaffold, is HA-based hydrogel functionalized to favor cell adhesion. For example, Ananthanarayanan studied HA gels of varying stiffness embedded with GBM spheroids and corroborated that their invasive capacity and morphological patterns were similar to what was seen *in vivo* in human brain slices, in opposition to glioma cells cultured in 2D and 3D collagen contexts (Ananthanarayanan et al., 2011). Differences were theorized to be related to the variation of expression of CD44. This was confirmed by Harley and co-workers, who identified CD44 as a key driver of glioma malignancy with cells encapsulated in gelatin and PEG-based hydrogels grafted with a HA hydrogel network (Pedron et al., 2013). Analogous observations were made by Erikson using porous chitosan–hyaluronic acid scaffolds of different stiffness, obtained varying the chitosan content. With a higher polymer content, the pore walls were thicker, with reduced interconnections between pores as well as the pore size (Erickson et al., 2018). Stiffness was shown to influence the morphology of the cell aggregates as well as the expression of drug resistance, hypoxia, and invasion-related genes (Mih et al., 2011; Zustiak et al., 2016; Erickson et al., 2018).

**Table 1:** A summary of various scaffolds used for glioblastoma responses.

**Scaffolds**	**Stiffness value range**	**Porosity**	**Cells**	**Behavior**	**References**
Chitosan/hyaluronic acid	Tunable between kPa to MPa	77.31 μm with 87.09% porosity; pore diameters between 134 to 179 μm	U-118 MG; GBM6 tumors; U87 MG	Tumor spheroid formation	(Florczyk et al., 2013; Cha et al., 2016; Kievit et al., 2016; Wang et al., 2016; Erickson et al., 2018)
Hyaluronic acid-methacrylate hydrogel	Ranging from 50 Pa to 35 kPa	Mesh sizes ranging from 50 to 150 nm	Human U373-MG and U87-MG; rat C6 glioma	GBM cell morphology and motility are regulated by stiffness. Different GBM invasiveness C6 > U87-MG > U373-MG	(Ananthanarayanan et al., 2011)
Gelatin methacrylate hydrogel	Ranging from 5 to 55 kPa	Micron scale larger	U87-MG	Biophysical regulation of GBM cell activity is not direct or clear	(Pedron and Harley, 2013)
Poly(ethylene-glycol) (PEG)-based hydrogels	Ranging from 1 to 26 kPa	–	U87-MG	Tumor spheroid formation	(Pedron et al., 2013; Wang et al., 2014)
Polyacrylamide hydrogels	Ranging from 0.2 to 50 kPa	–	Patient derived GBM cells (JK2, SJH1, WK1, RN1 and PR1)	Different migratory capacity. No detected association between cell morphology and migratory capacity	(Grundy et al., 2016)
Temperature responsive poly(N-isopropylacrylamide-co-Jeffamine M-1000^®;^ acrylamide)	Tunable between 153 and 1,240 Pa	–	Patient-derived GSC cell lines	On soft scaffolds (153–325 Pa), GSCs did not cluster into large neurosphere	(Heffernan et al., 2017)
Chitosan-alginate scaffold coated with hyaluronic acid	–	–	U-87 MG	Tumor spheroid formation	(Kievit et al., 2016)
GBM patient tissue derived ECM	78.09 ± 29.22 Pa	Porous and fibrous structure	Patient-derived GBM cells	GBM cells exhibited heterogeneous morphology and altered the invasion routes in a microenvironment-adaptive manner	(Koh et al., 2018)
Gelatin/alginate/fibrinogen hydrogel	–	–	GSC cell lines	GSC did not maintain their characteristics of cancer stem cells but showed differentiation potential	(Xingliang et al., 2016)
Collagen based hydrogel	Tunable stiffness	Tunable between 30 and 100 μm	U87, U251 and HS683 cell lines; primary glioma cells (OSU-2); patient derived GBM stem cells	Enhancement the malignancy of the glioma cells; spheroid formations	(Rao et al., 2013; Cha et al., 2016; Lv et al., 2016; Jia et al., 2018)

## Type of cells used in the *in vitro* models

A further critical key parameter is the choice of the cells used (Zustiak et al., 2014). Typically, commercially available human tumor cell lines are used, but they neglect predicting clinical outcomes due to different genetic aberrations. Glioblastoma stem-like cells (GSC) can mimic the tumor of origin being tumor cells with stem cell properties (Saha et al., 2008). However, they require isolating stem cells from each tumor patient and expansion to an adequate number within a clinically acceptable time. Aggregated cultures would recapitulate better the GBM microenvironment, allowing cell–cell contacts and collective migration. Non-adherent cultures lack the cell–matrix interactions present in the tumor stroma, whereas complex spherical cancer models (i.e. non-adherent cancer cell line-derived spheroids, or spheroids derived from primary tumor dissociation) can promote cell–cell interactions. The use of patient-derived cells cultured as neurospheres is a significant advance respect to glioma cell lines (Rao et al., 2013; Cha et al., 2016) however they do not accurately reproduce the original tumor composition due to heterogeneity loss and lack of an adhesive matrix. A solution could be represented by GBM organoids (Hubert et al., 2016), although these require months for generation, thus becoming useless in aid of patient treatment and not necessarily being an improvement to the patient outcomes (Oh et al., 2014), whereas neurosphere cultures can be established within only few weeks.

## Influence of compounding stimuli on the scaffolds

Diverging results have also been implicated with the matrix metalloprotease (MMP) secretion. For example, an increase of MMP-9 production in hyaluronic acid-based hydrogels with increasing stiffness was reported by Pedron's group while Wang and colleagues described an opposite behavior (Pedron et al., 2013, 2015; Wang et al., 2014). The reason, in our opinion, is related to the difficulty to discriminate the role of stiffness or of the biochemical stimuli on cell invasion. In fact, only a few works report a selected degree of decoupling of the mechanical properties, porosity, and/or biochemical cues. The interference of other compounding stimuli in the design of functional cell culture substrates should be minimized if not isolated. Changes in the ligand density and the pore size of the matrix may obstruct migration of cells and alter solute diffusion (Shu et al., 2002). For example, Cha used a different molecular weight of HA to simply coat the collagen I fibers without modifications and crosslinking (Cha et al., 2016). Using a higher molecular weight and 3D structure of the polymer may have induced different cell responses. Rao reported a high degree of thiolation, which may have altered the bioactivity of the substrate (Rao et al., 2013). Kumar and co-workers managed to decouple the effects by assembling hydrogel networks of collagen I and agarose and increasing the stiffness by increasing the concentration of non-adhesive agarose while keeping collagen I levels constant (Ulrich et al., 2010). However, increasing the concentration of agarose may result in smaller pores that restricted migration on stiffer hydrogels. Moreover, the presence of the agarose interfering with collagen fiber deformation and bundling may have thereby restricted local ability of tumor cells to stiffen their microenvironment (Kilian and Mrksich, 2012; Rape et al., 2015).

In conclusion there is still no existing artificial GBM microenvironment which can replace an *in vivo* model. It is essential to ask if it is worth to increase the complexity of the ECM microenvironment and to define which parameters are at least required to achieve a physiologically relevant model *ex vivo*. We think that tuning matrix stiffness will be pivotal at both a preclinical and clinical level, to move forward this field of investigation of cell behavior during tumorigenesis thereby providing an important tool to target and investigate the more effective therapy at different stages of cancer progression. To unravel the tumor invasiveness and to demonstrate their clinical value, a fully comprehensive analysis approach has to be achieved. This invites further study and highlights the importance of conducting parallel measurements using spheroid cell lines in highly multi-structured conditions as well as comparisons with patient outcomes.

## Author contributions

IP, SD, and BC wrote the manuscript. All authors reviewed the manuscript and have given approval to the final version of the manuscript.

### Conflict of interest statement

The authors declare that the research was conducted in the absence of any commercial or financial relationships that could be construed as a potential conflict of interest.
